# Efficacy of various sequences of transcatheter arterial chemoembolization combined with PD-1 inhibitors in advanced hepatocellular carcinoma: a retrospective analysis

**DOI:** 10.3389/fmed.2025.1574295

**Published:** 2025-06-18

**Authors:** Heping Zhu, Shenping Hu, Fuqiang Wang, Zhenyu Yin

**Affiliations:** ^1^Department of Hepatobiliary and Pancreatic Surgery, Xiamen Traditional Chinese Medicine Hospital, Xiamen, Fujian Province, China; ^2^Department of Radiation Oncology, Xiamen Women and Children’s Hospital, Xiamen, Fujian Province, China; ^3^Fujian Provincial Key Laboratory of Chronic Liver Disease and Hepatocellular Carcinoma, Department of Hepatobiliary Surgery, Zhongshan Hospital, Xiamen University, Xiamen, Fujian Province, China

**Keywords:** hepatocellular carcinoma, transcatheter arterial chemoembolization, PD-1 inhibitors, liver cancer, sequence

## Abstract

**Background:**

We aimed to explore whether the diverse sequences of Transcatheter Arterial Chemoembolization (TACE) combined with Programmed Death-1 (PD-1) inhibitors impact the prognosis of advanced hepatocellular carcinoma (HCC).

**Methods:**

In this single-center retrospective study, we collected data from patients with advanced HCC who underwent TACE combined with PD-1 inhibitors and categorized them into a group treated with PD-1 inhibitors after TACE (T+P) and a group treated with TACE after PD-1 inhibitors (P+T). Kaplan–Meier and logistic analyses were used to investigate the differences in treatment efficacy.

**Results:**

Ultimately, a total of 27 eligible patients were included in this study. Among them, 8 patients (29.6%) were in Barcelona Clinic Liver Cancer (BCLC) stage B, 19 patients were in stage C, 22 patients (81.5%) were in Child-Pugh stage A, five patients were in stage B,15 patients (55.6%) were in the P+T group, and 12 patients (44.4%) were in the T+P group. After a median follow-up of 5.0 months (1.8–17.3), all patients exhibited disease progression. According to the RECIST v1.1 criteria, the 6-month Disease Control Rate (DCR) in the T+P group and the P+T group was 58.3 and 20% (*p* = 0.048); the median Progression-Free Survival (PFS) in the two groups was 6.0 months (95% CI 5.32–6.67) and 4.2 months (95%CI 2.91–5.4) (HR, 2.59; 95% CI 1.10–6.10, *p* = 0.029).

**Conclusion:**

The effect of the T+P treatment was superior to that of the P+T treatment. Different sequences of TACE combined with PD-1 inhibitors influence the prognosis of patients with advanced HCC.

## Introduction

Primary liver cancer is one of the most common and fatal malignancies worldwide. Its global incidence rate ranks sixth among all tumors, and its mortality rate ranks third, with more than 80% of patients diagnosed with Hepatocellular carcinoma (HCC) (1). Due to the relatively insidious onset, lack of effective screening, and early diagnostic methods, 70–80% of HCC patients are diagnosed at an advanced stage, with a five-year survival rate of less than 15% and an overall survival of only 6–20 months (2, 3). Therefore, novel therapeutic approaches are necessary. In 2007, the SHARP and Asia-Pacific trials successively confirmed the role of sorafenib in treating advanced HCC. However, owing to its high adverse event rate and low treatment response rate, the emergence of sorafenib failed to completely change the challenging treatment situation of advanced HCC (4, 5). In 2012, immune checkpoint inhibitor (ICIs) therapies targeting programmed cell death (PD-1), programmed cell death ligand 1, and cytotoxic T Lymphocyte Associate Protein-4 were successful in the treatment of solid tumors, such as melanoma and lung cancer (6, 7). Subsequently, ICIs, such as pembrolizumab, tislelizumab, and camrelizumab, have been successively confirmed to be safe and effective in the treatment of HCC (8–10).

Transcatheter arterial chemoembolization (TACE) is an important treatment for HCC, not only to directly eliminate tumor cells but also to promote the release of tumor-specific antigens to enhance the anti-tumor immune effect (11). In a study of Gypican-3(GPC3)-mediated specific T cell immune response, 55% of HCC patients exhibited increased GPC3-specific cytotoxic T lymphocytes in blood circulation after receiving TACE treatment, while only 11% of patients showed increased GPC3-specific cytotoxic T lymphocytes in circulation after surgical resection (12).

TACE combined with PD-1 inhibitors is a better treatment option for advanced HCC, but whether sequencing affects the efficacy and safety of the combination remains to be further investigated. Therefore, we compared the clinical efficacy of patients who received PD-1 inhibitor therapy after TACE with those who received PD-1 inhibitor therapy before TACE to observe whether the treatment sequence had an impact on prognosis.

## Materials and methods

### Patient cohorts

The clinical data of patients with advanced HCC who underwent TACE combined with PD-1 inhibitors between June 2019 and June 2022 were retrospectively analyzed.

**Inclusion Criteria:** Age ranging from 30–80 years old; patients receiving TACE combined with PD-1 inhibitors; according to the RECIST 1.1 standard, there is at least one evaluable lesion; BCLC B/C stage; Eastern Cooperative Oncology Group (ECOG) ranging from 0 to 2; Child-Pugh score A/B stage.

**Exclusion Criteria:** Missing case data; Use of other treatment methods that may affect prognosis during the treatment period, such as molecular targeted drugs, systemic radiotherapy and chemotherapy, and palliative surgical treatment; Expected lifespan ≤ 3 months; patients with major diseases of other major organ systems, such as those affected by other malignant tumors, infectious diseases, blood diseases, severe liver and kidney dysfunction, and other malignant diseases.

### Methods

**Figure 1:**
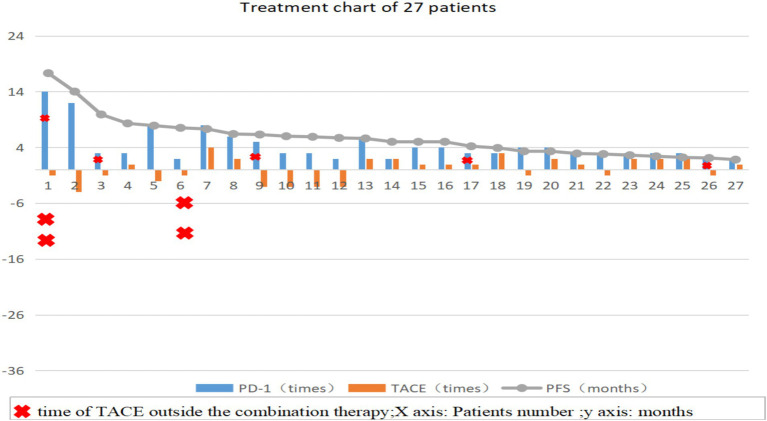
Treatment process diagram of 27 patients, the number of TACE procedures in the T+P group is represented as a negative value. TACE: Transcatheter arterial chemoembolization; PD-1: Programmed death receptor-1; PFS: Progression-Free survival.

All patients were evaluated based of tumor condition and physical tolerance, and TACE was performed by the same clinician. Twelve (44.4%) patients received PD-1 inhibitor treatment after completing TACE (T+P group); if no obvious active lesions appeared, only PD-1 inhibitor treatment was administered during each hospitalization. Fifteen patients (55.6%) received PD-1 inhibitor treatment first (P+T group) and received TACE within 1–2 weeks. If no obvious active lesions were observed after evaluation, only PD-1 inhibitor treatment was administered during each hospitalization (Figure 1).

The PD-1 inhibitors used by the patients included camrelizumab (12 cases, 44.4%), toripalimab (7 cases, 25.9%), sintilimab (3 cases, 11.1%), pembrolizumab (2 cases, 7.4%), Tislelizumab (2 cases, 7.4%), nivolumab (1 case, 3.7%). Intravenous infusion was administered once every 3 weeks, and the dose was calculated according to the drug instructions (camrelizumab 200 mg/time, sintilimab 200 mg/time, toripalimab 240 mg/time, nivolumab 180 mg/time, pembrolizumab 100 mg/time, Tislelizumab 200 mg/time) until the disease was evalu-ated as progression, occurrence of treatment-related serious adverse events or the patient refused to continue the current treatment plan.

#### Efficacy and safety assessment

Relevant hematological test and imaging examination data of patients before each hospitalization and during treatment were collected, including: detailed medical history and physical examination of patients, imaging examinations including chest CT, abdominal/liver dynamic enhanced CT or MRI examination, hepatobiliary color Doppler ultrasound, bone ECT, plain scan of brain CT, etc. Patients underwent liver enhanced CT/MRI examination every 1–2 months. If the lesion remained stable, the review interval could be appropriately extended. If there were signs of disease progression, the review interval was shortened; Laboratory tests included blood routine, coagulation function, alpha-fetoprotein (AFP), liver and kidney function, myocardial enzymes, thyroid function, urine analysis, and stool routine.

#### Efficacy evaluation

Evaluation was performed according to the RECIST 1.1 standard: Measured by experienced radiologists and hepatobiliary surgeons based on image data, and classified according to the measurement results as:

**Complete Response (CR):** All target lesions except nodular diseases completely disappear. All target nodules must shrink to normal size (short axis < 10 mm).
**Partial Response (PR):** The sum of the diameters of all measurable target lesions is reduced by ≥ 30% from the baseline.
**Progressive Disease (PD):** Taking the minimum sum of the diameters of all measured target lesions during the entire experimental study as a reference, the diameter sum is increased by at least 20% (if the baseline measurement value is the minimum, the baseline value is used as a reference); in addition, the absolute value of the diameter sum must be increased by at least 5 mm (the appearance of one or more new lesions is also regarded as disease progression).
**Stable Disease (SD):** The degree of reduction of the target lesion does not reach PR, and the degree of increase does not reach the PD level, which is between the two.

Overall Response Rate (ORR) is calculated as (CR + PR) / total number of cases * 100%.

Disease Control Rate (DCR) is calculated as (CR + PR + SD) / total number of cases * 100%.

Progression-Free Survival (PFS) is the interval from the time when the patient first received PD-1 inhibitor treatment to disease progression or death for any reason.

#### Adverse events

According to the common terminology criteria for adverse events (version 5.0).

#### Statistical analysis

SPSS software (version 26.0) was used for the statistical analysis. Count data were expressed as cases or rates, and the Chi-square test was used for comparison between groups. For measurement data that conformed to the normal distribution, these were expressed as mean ± SD, and for measurement data that did not conform to the normal distribution, the median (interquartile range) was used. Kaplan–Meier and COX regression were used to analyze the median PFS. Binary Logistic regression was used to compare the differences in DCR. *p* < 0.05 was considered statistically significant.

## Results

### Patient characteristics

**Figure 2:**
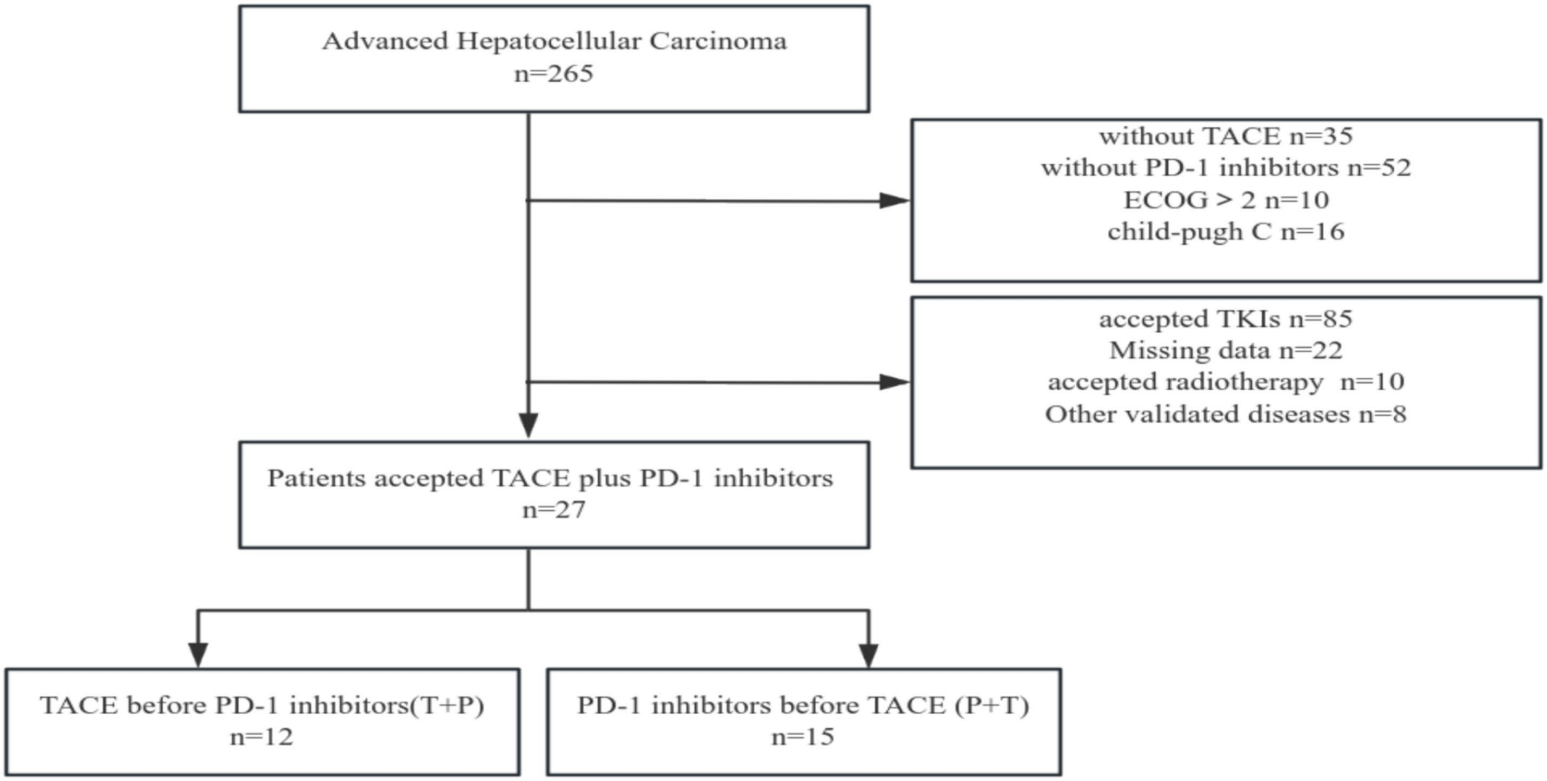
Flow diagram for enrolling patients in this study. TACE: Transcatheter arterial chemoembolization; PD-1: Programmed death receptor-1; TKIs: Tyrosine kinase inhibitors.

In total, 265 eligible patients with advanced HCC were screened. Finally, 27 patients were enrolled in the study (Figure 2). Two patients received additional TACE before combined treatment, and five patients received additional TACE during combined treatment due to suspected residual active tumor foci. Among them, 24 patients were male (88.9%) and 3 were female (11.1%); 8 patients (29.6%) were in BCLC stage B and 19 patients (70.4%) were in stage C; 26 patients (96.3%) were infected with hepatitis B virus; 9 patients (33.3%) had alpha-fetoprotein levels lower than 400 ng/mL; 9 patients (33.3%), 12 patients (44.4%), and 6 patients (22.2%) had ECOG performance statuses of 0, 1, and 2, respectively; 22 patients (81.5%) were in Child-Pugh stage A and 5 patients (18.5%) were in stage B; 7 patients (25.9%) had extrahepatic metastasis and 20 patients (74.1%) did not; 14 patients (51.9%) had invasion of the hepatic vein, inferior vena cava, and portal vein branches; 9 patients (33.3%) had less than 4 tumors and 18 patients (66.7%) had more than 3 tumors; 13 patients (48.1%) had tumor diameters less than 10 cm, and 14 patients (51.9%) had diameters ≥ 10 cm; 15 patients (55.6%) received PD-1 inhibitor treatment first and then TACE (P+T), while 12 patients (44.4%) received TACE first and then PD-1 inhibitor treatment (T+P).

### Efficacy and adverse reactions

**Figure 3:**
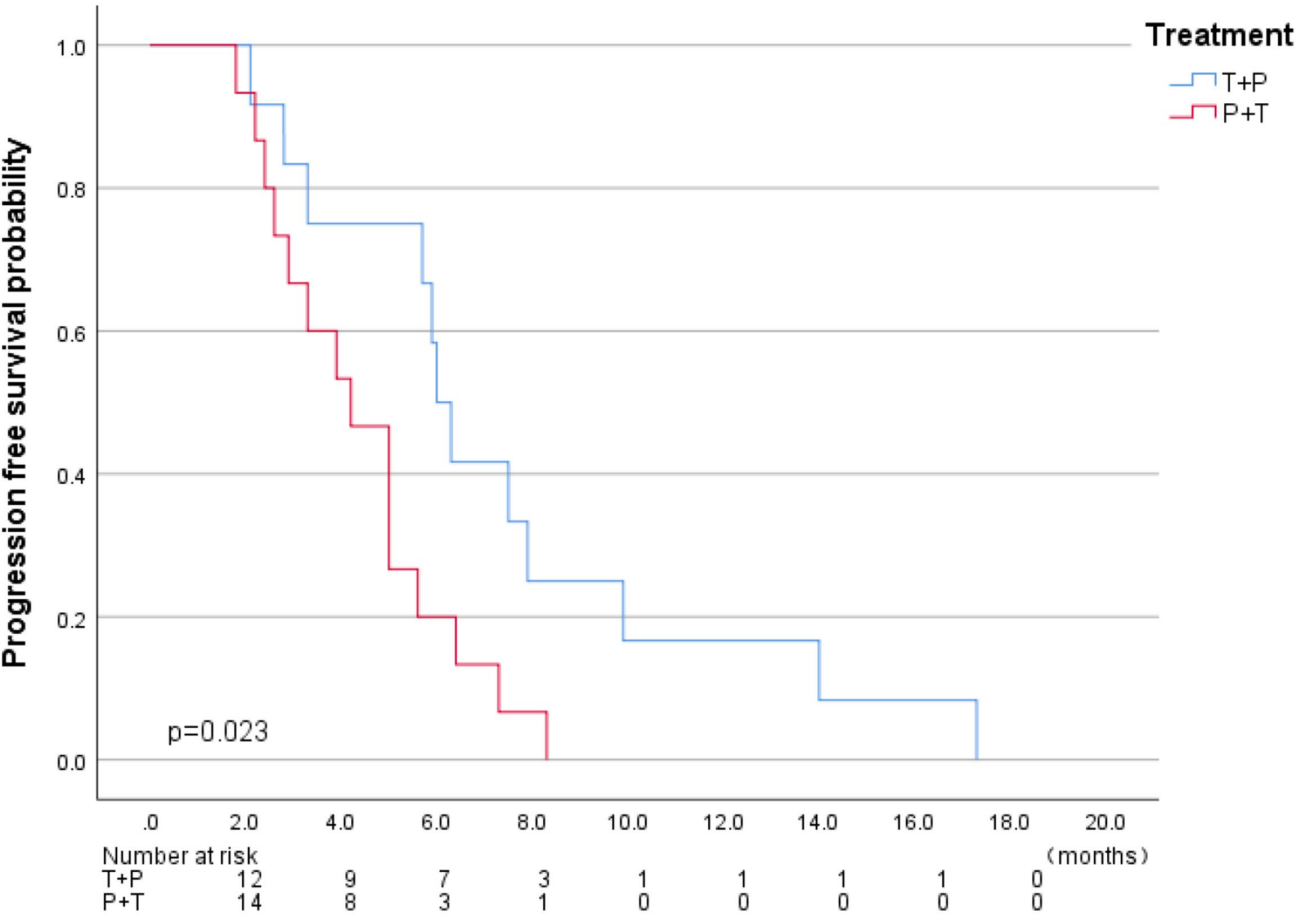
Kaplan-Meier analysis of median PFS in two groups under the RECIST v1.1criteria.

The median follow-up time was five months (1.8–17.3), and all patients experienced disease progression during the follow-up period. We analyzed the baseline data between the two treatment groups using the Chi-square test, and found no significant differences in staging, sex, age, major vascular invasion, or alpha-fetoprotein levels between the two groups. The baseline characteristics of two groups were present in Table 1. In terms of 6-month DCR, the T+P group and the P+T group exhibited rates of 58.3 and 20% (*p* = 0.048) (Table 2); Kaplan–Meier analysis showed a significant difference in the median PFS between the two groups, which was 6.0 months (95% CI 5.32–6.67) for the T+P group and 4.2 months for the P+T group (95% CI 2.91–5.4) (HR, 2.59; 95% CI 1.10–6.10, *p* = 0.029) (Figure 3). Overall, the T+P combination was significantly more effective than the P+T combination in terms of short-term efficacy. In terms of adverse reactions, 18 patients in this study experienced at least one treatment-related adverse event, mainly manifested as liver function damage and abdominal pain. Four patients exhibited grade 3 adverse reactions, which were all relieved after symptomatic and supportive treatment. The total incidence of adverse events in both treatment groups was 66.7% (Table 3).

**Table 1:** Baseline characteristics in two groups.

Variables	Overall (*N* = 27)	TACE+PD-1 Inhibitor(*N* = 12)	PD-1 Inhibitor+TACE(*N* = 15)	*p*
Gender (*n*/%)				
Male	24(88.9%)	10(83.3%)	14(93.3%)	0.569
Female	3(11.1%)	2(16.7%)	1(6.7%)	
Age (years)				
<60	19(70.4%)	9(75.0%)	10(66.7%)	0.696
≥60	8(29.6%)	3(25.0%)	5(33.3%)	
Child-Pugh class				
A	22(81.5%)	10(83.3%)	12(80.0%)	1.000
B	5(18.5%)	2(16.7%)	3(20.0%)	
ECOG performance				
0	9(33.3%)	5(41.6%)	4(26.7%)	0.261
1	12(44.4%)	6(50.0%)	6(40.0%)	
2	6(22.2%)	1(8.4%)	5(33.3%)	
Hepatitis B virus antigens				
Negative	1(4.0%)	0(0.0%)	1(6.7%)	1.000
Positive	26(96.0%)	12(100.0%)	14(93.3%)	
Alpha fetoprotein (ng/mL)				
<400	9(33.3%)	5(41.7%)	4(26.7%)	0.448
≥400	18(66.7%)	7(58.3%)	11(73.3%)	
Number of tumors				
≤3	9(33.3%)	6(50.0%)	3(20.0%)	0.127
>3	18(66.7%)	6 (50.0%)	12(80.0%)	
Largest diameter of tumors(cm)				
<10	13(48.1%)	5(41.7%)	8(53.3%)	0.704
≥10	14(51.9%)	7(58.3%)	7(46.7%)	
Extrahepatic metastasis				
No	20(74.1%)	9(75.0%)	11(73.3%)	1.000
Yes	7(25.9%)	3(25.0%)	4(26.9%)	
Vascular invasion				
No	13(48.1%)	8(75.0%)	5(33.3%)	0.128
Yes	14(51.9%)	4(25.0%)	10(66.7%)	
BCLC stage				
B	8(29.6%)	5(41.7%)	3(20.0%)	0.398
C	19(70.4%)	7(58.3%)	12(80.0%)	

TACE: Transcatheter arterial chemoembolization; PD-1: Programmed death receptor-1; ECOG: Eastern cooperative oncology group; BCLC: Barcelona clinic liver cancer.

**Table 2:** Tumor response in two groups.

Efficacy evaluation	TACE+PD-1 Inhibitor(*N* = 12)	PD-1 Inhibitors+TACE(*N* = 15)	*p*
CR	2(16.7%)	0(0%)	
PR	3(25.0%)	2(13.3%)	
SD	2(16.7%)	1(6.7%)	
DCR	7(58.3%)	3(20.0%)	0.048

TACE: Transcatheter arterial chemoembolization; PD-1: Programmed death receptor-1; CR: Complete response; PR: Partial response; SD: Stable disease; DCR: Disease control rate.

**Table 3:** Adverse events in two groups.

Adverse events	TACE+PD-1 Inhibitor(*N* = 12)		PD-1 Inhibitor+TACE(*N* = 15)	
	Any grade	Grade 3–4	Any grade	Grade 3–4
Overall	8(66.7%)	2(16.7%)	10(66.7%)	2(13.3%)
Fatigue	3(25.0%)	0(0%)	5(33.3%)	0(0%)
Hand-foot skin reaction	3(25.0%)	0(0%)	4(26.7%)	0(0%)
Abnormal liver function	8(66.7%)	2(16.7%)	9(60.0%)	2(13.3%)
Hypothyroidism	2 (16.7%)	0(0%)	4(26.7%)	0(0%)
Decreased appetite	6(50.0%)	0(0%)	6(40.0%)	0(0%)
hematotoxicity	2(16.7%)	0(0%)	3(20.0%)	0(0%)
Ventosity	1(8.3%)	0(0%)	3(20.0%)	0(0%)
Abdominal pain	8(66.7%)	0(0%)	8(53.3%)	0(0%)

TACE: Transcatheter arterial chemoembolization; PD-1: Programmed death receptor-1.

## Discussion

TACE is the main treatment for advanced HCC, but its efficacy is limited. Checkmate 459 and Keynote-240 confirmed that PD-1 inhibitors improved the treatment response rate and progression-free survival of patients with advanced HCC (13, 14). NCT01853618 and NCT02821754 preliminarily verified the efficacy and safety of TACE combined with PD-1 inhibitors for the treatment of advanced HCC. However, in previous clinical trials, few researchers compared the impact of the treatment sequence of TACE combined with PD-1 inhibitors on efficacy. In this study, patients who received TACE treatment first (T+P group) exhibited a longer median PFS of 6.0 months compared to 4.2 months in the P+T group (*p* = 0.029), and the 6-month DCR was 58.3 and 20% (*p* = 0.048). This indicates that changing the sequence of combination therapy may affect the final efficacy. This may be related to the influence of TACE on immune system activity. Adam et al. suggested that TACE treatment induces a hypoxic environment in the liver, resulting in upregulated expression of HIF-1a, increased expression of PD-L1 on the surface of immune and tumor cells, and immunosuppression (15); Akizuki discovered as early as the end of the last century that peripheral blood γδT cells decreased significantly after TACE treatment, and the body’s anti-tumor activity was weakened (16); Tan et al. confirmed through single-cell gene sequencing technology that the number of TREM2 + TAMs in the tumor microenvironment after TACE surgery increased significantly and inhibited the activity of CDCD8 + T cells (17). These studies suggest that the immune function of the patients is inhibited to a certain extent after TACE. PD-1 inhibitors restore the ability of immune cells, such as CD4 + T cells, CD8 + T cells, and natural killer cells, to recognize and kill tumor cells by blocking the binding of PD-1 to the membrane of immune cells and PD-L1 expressed on the membrane of tumor cells (18). If TACE treatment is received shortly after PD-1 inhibitor treatment, will the activated immune cells and immune functions be weakened due to TACE treatment? In this study, the PFS of patients who received T+P treatment was significantly better than that of patients who received P+T treatment. This may be because the immune function activated after PD-1 inhibitor treatment was interfered with by TACE treatment and the efficacy of immunotherapy was not maximized, thereby adversely affecting the effect of the combination treatment. In the T+P treatment mode, immune activity activated by the PD-1 inhibitor was not affected by previous TACE treatments. In contrast, the use of PD-1 inhibitors may reverse the potential immunosuppressive effect of TACE, thereby generating a positive synergistic effect of the combination treatment, ultimately benefiting patients. However, the combination treatment sequence that can promote rather than inhibit immune balance in the treatment process and ultimately be beneficial to the prognosis of patients requires further exploration.

## Conclusion

This retrospective analysis suggested that the sequence of TACE and PD-1 inhibitors may influence patient outcomes, with TACE followed by PD-1 inhibitors showing better efficacy. These findings need to be confirmed in larger prospective clinical trials.

Although this study is clinically significant, limitations include its single-center retrospective nature, small sample size, and potential bias due to unrecorded adverse events. Further research is required to validate these findings.

## Data Availability

The raw data supporting the conclusions of this article will be made available by the authors, without undue reservation.
